# Changes in demographics, treatment and outcomes in a consecutive cohort who underwent transcatheter aortic valve implantation between 2005 and 2020

**DOI:** 10.1007/s12471-022-01662-2

**Published:** 2022-02-25

**Authors:** M. J. A. G. De Ronde-Tillmans, R. M. Nuis, J. A. Goudzwaard, P. A. Cummins, T. W. Hokken, M. P. H. Van Wiechen, J. F. W. Ooms, J. Daemen, N. M. D. A. Van Mieghem, F. U. S. Mattace-Raso, M. J. Lenzen, P. P. T. de Jaegere

**Affiliations:** 1 Department of Cardiology, Erasmus University Medical Centre, Rotterdam, The Netherlands; 2 Department of Internal Medicine, Section of Geriatrics, Erasmus University Medical Centre, Rotterdam, The Netherlands

**Keywords:** Aortic stenosis, Transcatheter aortic valve implantation, Survival, Clinical outcomes

## Abstract

**Introduction:**

Transcatheter aortic valve implantation (TAVI) has matured to the treatment of choice for most patients with aortic stenosis (AS). We sought to identify trends in patient and procedural characteristics, and clinical outcomes in all patients who underwent TAVI between 2005 and 2020.

**Methods:**

A single-centre analysis was performed on 1500 consecutive patients who underwent TAVI, divided into three tertiles (T) of 500 patients treated between November 2005 and December 2014 (T1), January 2015 and May 2018 (T2) and June 2018 and April 2020 (T3).

**Results:**

Over time, mean age and gender did not change (T1 to T3: 80, 80 and 79 years and 53%, 55% and 52% men, respectively), while the Society of Thoracic Surgeons risk score declined (T1: 4.5% to T3: 2.7%, *p* < 0.001). Use of general anaesthesia also declined over time (100%, 24% and 1% from T1 to T3) and transfemoral TAVI remained the default approach (87%, 94% and 92%). Median procedure time and contrast volume decreased significantly (186, 114 and 56 min and 120, 100 and 80 ml, respectively). Thirty-day mortality (7%, 4% and 2%), stroke (7%, 3% and 3%), need for a pacemaker (19%, 22% and 8%) and delirium (17%, 12% and 8%) improved significantly, while major bleeding/vascular complications did not change (both approximately 9%, 6% and 6%). One-year survival was 80%, 88% and 92%, respectively.

**Conclusion:**

Over our 15 years’ experience, patient age remained unchanged but the patient risk profile became more favourable. Simplification of the TAVI procedure occurred in parallel with major improvement in outcomes and survival. Bleeding/vascular complications and the need for pacemaker implantation remain the Achilles’ heel of TAVI.

**Supplementary Information:**

The online version of this article (10.1007/s12471-022-01662-2) contains supplementary material, which is available to authorized users.

## What’s new?

- The first transcatheter aortic valve implantation (TAVI) procedure in the Netherlands was performed on 15 November 2005 at the Erasmus University Medical Centre, Rotterdam, using general anaesthesia, surgical access and circulatory support.
- Elimination of circulatory support and replacement of surgical access by echo-guided access led to a true percutaneous approach.
- The TAVI Care & Cure clinical research programme was initiated in October 2013 and included a team of geriatricians enhancing the quality of treatment decisions and the prevention of delirium.
- Filter-based cerebral embolic protection preventing peri-operative stroke was introduced in January 2013.
- TAVI procedures were simplified further by eliminating general anaesthesia (PCI-like procedure, August 2015).

## Introduction

Since the first transcatheter aortic valve implantation (TAVI) was performed in a patient with aortic stenosis (AS) at prohibitive surgical risk in 2002, TAVI has evolved into a standardised minimally invasive treatment for high-risk patients [1–3]. Expanding operator experience, improvements in catheter and valve design and progress in peri-procedural patient care have contributed to improved outcomes and have been followed by further simplification of the TAVI pathway (i.e. post-procedural monitoring and recovery on the cardiology ward in lieu of the intensive care unit, early mobilisation and early discharge) [4–7].

As randomised controlled trials (RCTs) showed equivalence between TAVI and surgical aortic valve replacement (SAVR), the European Society of Cardiology updated the guidelines in 2017 by expanding indications for TAVI in patients at intermediate surgical risk [8]. Importantly, a recent RCT showed transfemoral TAVI to be superior to SAVR in low-risk patients [9].

In the context of the above, we sought to analyse whether this development had an impact on the patients that we accepted and treated for TAVI in conjunction with in-hospital outcomes and survival post-discharge of the first 1500 patients that underwent TAVI in our institution between 2005 and 2020.

## Methods

### Study population

The study population comprised the first 1500 patients with AS (including 15 patients with aortic regurgitation more severe than stenosis) who underwent TAVI between November 2005 and April 2020 at the Erasmus University Medical Centre (Erasmus MC), Rotterdam, The Netherlands. Patient selection and treatment strategy was based on clinical (i.e. age, comorbidities, surgical risk) and anatomical characteristics (access-site suitability) in the context of available valve technology per studied time period. Self-expanding valves were primarily used during the start-up phase in 2005–2007, after which self-expanding, balloon-expandable and mechanically expanded (the last-mentioned since 2013) devices were used in more recent phases of the TAVI programme. In the initial period (November 2005–October 2010), the final treatment decision was based on arbitrary case discussions primarily between an interventional cardiologist and a thoracic surgeon, complemented with a radiologist with valvular access and closure expertise. From October 2010, patient eligibility for TAVI, choice of treatment and strategy were decided during a structured weekly meeting of a multidisciplinary heart team consisting of an interventional cardiologist, a cardiac surgeon, a cardiac anaesthetist and an imaging cardiologist [10]. From October 2013, all patients with AS evaluated at the outpatient cardiology clinic were also seen by the geriatrician, who assessed specific pre-defined geriatric domains such as physical function, frailty and cognitive status. This was done in the framework of the TAVI Care & Cure clinical research project and programme, the details of which have been described before [11]. A diagnosis of delirium was based on geriatric assessment as described previously [12]. All other endpoint definitions are in accordance with VARC‑2 criteria [13].

The study has been reviewed and approved by the ethics committee of the Erasmus MC (MEC-2014-277) and was conducted according to the Erasmus MC regulations for appropriate use of data in patient-oriented research and the privacy policy of the Erasmus MC. Data on mortality after hospital discharge were collected via the Dutch Civil Registry.

### Statistical analyses

For the assessment of changes in patient demographics and clinical outcomes, the study population of 1500 patients was categorised into three chronological tertiles of 500 patients each. Tertile 1 (T1) included all TAVI procedures performed between November 2005 and December 2014; tertile 2 (T2) included patients treated between January 2015 and May 2018, while tertile 3 (T3) included patients treated between June 2018 and April 2020.

Continuous variables are presented as mean ± standard deviation or median with interquartile range, and differences between the three tertiles were analysed by one-way ANOVA or Kruskal-Wallis test, as appropriate. Categorical variables are presented as numbers and percentages and were analysed by chi-square test or Fisher’s exact test, as appropriate. One-year survival was studied with the Kaplan-Meier method; the log-rank test was used to evaluate differences between tertiles. Results are assumed to be statistically significant if *p* < 0.05. All data were analysed with SPSS software (SPSS Version 25; IBM Corp., Armonk, NY, USA).

## Results

The study results (baseline characteristics, procedural details, in-hospital outcomes and mortality at 30 days) are summarised in Tab. 1, 2 and 3. The first 500 patients were treated in the first 9 years and 2 months (November 2005–December 2014), while the next two cohorts of 500 patients were treated in the following 3 years and 5 months (January 2015–May 2018) and 1 year and 10 months (June 2018–March 2020). In other words, it took almost 10 years to include the first 500 patients but only 3.5 years and less than 2.0 years for the subsequent cohorts.

**Table 1:** Demographics and baseline characteristics

	Total cohort	Tertile 1	Tertile 2	Tertile 3	
Period of admission	Nov 2005–Apr 2020	Nov 2005–Dec 2014	Jan 2015–May 2018	Jun 2018–Apr 2020	
	*n* = 1500	*n* = 500	*n* = 500	*n* = 500	*p*-value
Age, mean ± SD	79.4 ± 7.9	79.8 ± 8.0	79.5 ± 7.8	78.9 ± 7.8	0.17
Male, *n* (%)	797 (53)	263 (53)	273 (55)	261 (52)	0.72
Body mass index, mean ± SD	27.0 ± 5.0	26.5 ± 4.6	27.1 ± 4.9	27.3 ± 5.4	** 0.027**
Medical history, *n* (%)					
– Stroke	318 (21)	104 (21)	101 (20)	113 (22)	0.63
– Myocardial infarction	308 (21)	125 (25)	106 (21)	77 (15)	** 0.004**
– Peripheral vascular disease	617 (41)	241 (48)	218 (44)	158 (32)	**<** **0.001**
– Percutaneous coronary intervention	439 (29)	146 (29)	161 (32)	132 (27)	0.14
– Coronary artery bypass graft surgery	263 (18)	115 (23)	86 (17)	62 (12)	**<** **0.001**
– Previous valve replacement	88 (6)	26 (5)	31 (6)	31 (6)	0.74
Risk factors, *n* (%)					
– Diabetes mellitus	460 (31)	150 (30)	159 (32)	151 (30)	0.65
– Hypertension	1099 (73)	345 (69)	390 (78)	364 (73)	** 0.002**
– Chronic renal failure	499 (33)	138 (28)	198 (40)	163 (33)	** 0.001**
– Chronic obstructive pulmonary disease	306 (20)	133 (27)	93 (19)	80 (16)	**<** **0.001**
Malignancy					0.47
– Curative treatment	263 (18)	76 (15)	96 (19)	91 (18)	
– Under treatment or proven metastases	69 (5)	20 (4)	22 (4)	27 (5)	
Clinical characteristics, *n* (%):					
– Pacemaker	149 (10)	47 (9)	50 (10)	52 (11)	0.68
– LBBB/RBBB, *n*/*n* (total %)	172/137 (21)	69/44 (14)	44/50 (19)	59/43 (20)	0.25
– Porcelain aorta	42 (3)	15 (3)	16 (3)	11 (2)	0.60
– Chronic haemodialysis	32 (2)	16 (3)	11 (2)	5 (1)	0.055
– Creatinine, median (IQR)	95 (76–120)	96 (77–123)	96 (75–123)	93 (74–115)	0.057
– Haemoglobin, mean ± SD	7.7 ± 1.1	7.7 ± 1.0	7.8 ± 1.0	7.7 ± 1.1	0.14
NYHA class III/IV	1000 (67)	403 (81)	307 (61)	290 (58)	**<** **0.001**
STS score, median (IQR)	3.9 (2.5–5.8)	4.5 (3.3–6.5)	4.3 (2.9–6.7)	2.7 (1.8–4.2)	**<** **0.001**
Echocardiographic parameters					
– Aortic valve area (cm^2^)^a^, median (IQR)	0.79 (0.60–0.90)	0.70 (0.60–0.90)	0.74 (0.60–0.90)	0.80 (0.68–0.90)	**<** **0.001**
– Mean aortic gradient (mm Hg)^b^, median (IQR)	39 (30–49)	41 (30–52)	38 (30–48)	38 (30–48)	** 0.010**
– AR ≥ moderate, *n* (%)^c^	255 (19)	77 (19)	91 (19)	87 (19)	0.99
– MR ≥ moderate, *n* (%)^d^	308 (23)	79 (20)	130 (27)	99 (21)	** 0.015**
Indication for TAVI*, n* (%)					0.061
– Severe native AS	1386 (92)	466 (93)	448 (90)	472 (94)	
– Severe native AR	15 (1)	4 (1)	7 (1)	4 (1)	
– Degenerated surgical bio-prosthesis	37 (2.6)	13 (3)	19 (4)	5 (1)	
– Other (i.e. moderate AS, mixed AS/AR)	62 (4)	17 (3)	26 (5)	19 (4)	

*LBBB* Left bundle branch block, *RBBB* right bundle branch block, *IQR* interquartile range, *NYHA* New York Heart Association, *STS* Society of Thoracic Surgeons, *AR* aortic regurgitation, *AS* aortic stenosis, *MR* mitral regurgitation, *TAVI* transcatheter aortic valve implantation

Data not available in ^a^121 patients, ^b^121 patients, ^c^170 patients and ^d^149 patients

**Table 2:** Procedural characteristics

	Total cohort	Tertile 1	Tertile 2	Tertile 3	
Period of admission	Nov 2005–Apr/2020	Nov 2005–Dec 2014	Jan 2015–May 2018	Jun 2018–Apr 2020	
	*n* = 1500	*n* = 500	*n* = 500	*n* = 500	*p*-value
Urgent procedure, *n* (%)	52 (4)	15 (3)	15 (3)	22 (4)	0.38
PCI (combined), *n* (%)	158 (11)	45 (9)	49 (10)	64 (13)	0.077
Anaesthesia, *n* (%)^a^					**<** **0.001**
– General anaesthesia	622 (42)	500 (100)	117 (24)	5 (1)	
– Local anaesthesia	861 (57)	0	377 (75)	484 (97)	
– Conscious sedation	11 (1)	0	6 (1)	5 (1)	
Access, *n* (%)^a^					**<** **0.001**
– Transfemoral	1360 (91)	434 (87)	468 (94)	458 (92)	
– Transapical	43 (3)	29 (6)	14 (3)	0 (0)	
– Subclavian	44 (3)	6 (1)	1 (0)	37 (7)	
– Other (i.e. axillary, aortic)	50 (3)	31 (6)	17 (3)	2 (0.4)	
Cerebral protection device, *n* (%)	604 (40)	129 (26)	260 (52)	215 (43)	**<** **0.001**
Balloon pre-dilatation, *n* (%)	598 (40)	418 (84)	57 (11)	123 (25)	**<** **0.001**
Balloon post-dilatation, *n* (%)	381 (26)	93 (19)	138 (28)	150 (30)	**<** **0.001**
Prosthesis type, *n* (%)^a^					**<** **0.001**
– Self-expanding^b^	827 (55)	354(71)	222 (45)	251 (51)	
– Mechanically expanded^c^	208 (14)	41 (8)	118 (24)	49 (10)	
– Balloon expandable^d^	460 (31)	105 (21)	159 (32)	196 (40)	
Valve-in-valve, *n* (%)	39 (3)	20 (4)	8 (2)	11 (2)	0.11
Conversion to SAVR, *n* (%)	5 (0.3)	1 (0)	2 (0)	2 (0)	0.80
Vascular closure, *n* (%)					**<** **0.001**
– Surgical	422 (28)	390 (78)	21 (4)	11 (2)	
– Prostar XL	77 (5)	59 (12)	18 (4)	0	
– Proglide	318 (21)	43 (9)	131 (26)	144 (29)	
– Manta	571 (38)	0	229 (46)	342 (68)	
– Other (i.e. TR band, left in situ)	112 (8)	8 (2)	101 (20)	3 (1)	
Fluoroscopy time (min), median (IQR)	15 (11–21)	21 (16–31)	15 (12–20)	13 (10–18)	**<** **0.001**
Contrast volume (ml), median (IQR)	100 (80–130)	120 (95–170)	100 (80–125)	80 (75–100)	**<** **0.001**
Procedure time (min), median (IQR)	120 (65–177)	186 (156–224)	114 (80–149)	56 (43–72)	**<** **0.001**

*PCI* Percutaneous coronary intervention, *SAVR* surgical aortic valve replacement, *IQR* interquartile range

^a^Due to missing data (≤ 6 cases), totals may not add up to 1500/500

^b^Includes: Medtronic CoreValve, Evolut R, Evolut Pro, Symetis Acurate, Edwards Centera, JenaValve and St Jude Medical Portico

^c^Includes: LOTUS, LOTUS Edge and LOTUS Depth Guard (Boston Scientific)

^d^Includes: Edwards XT and Edwards Sapien 3

**Table 3:** Clinical outcomes

	Total cohort	Tertile 1	Tertile 2	Tertile 3	
Period of admission	Nov 2005–Apr 2020	Nov 2005–Dec 2014	Jan 2015–May 2018	Jun 2018–Apr 2020	
	*n* = 1500	*n* = 500	*n* = 500	*n* = 500	*p*-value
All-cause mortality,* n *(%)					
– 30-day mortality	63 (4)	34 (7)	18 (4)	11 (2)	0.001
– 1-year mortality	201 (14)	100 (20)	62 (12)	39^a^	**<** **0.001**
– 3-year mortality	370 (28)	187 (38)	131^b^	52^b^	**<** **0.001**
In-hospital complications,* n *(%)					
– All bleeding complications (< 24 h)	205 (14)	73 (15)	75 (15)	57 (11)	0.19
a. Life-threatening/disabling/major	105 (7)	46 (9)	28 (6)	31 (6)	0.057
– Peri-procedural myocardial infarction (< 72 h)	13 (1)	4 (1)	3 (1)	6 (1)	0.58
– Stroke/transient ischaemic attack	63 (4)	35 (7)	15 (3)	13 (3)	** 0.001**
– All vascular complications	263 (18)	82 (16)	96 (19)	85 (17)	0.47
a. Major vascular complication	121 (8)	43 (9)	39 (8)	40 (8)	0.35
– Delirium	123 (12)	37 (17)	59 (12)	27 (8)	** 0.018**
– Conduction disorders, *n* (%)					
a. New left bundle branch block	744 (56)	256 (68)	268 (58)	220 (46)	**<** **0.001**
b. 3rd-degree AV block	147 (10)	46 (9)	65 (13)	36 (7)	** 0.007**
c. Temporary pacemaker, *n* (%)	666 (46)	300 (60)	309 (62)	57 (13)	**<** **0.001**
d. New permanent pacemaker^c^, *n* (%)	224 (17)	87 (19)	99 (22)	38 (8)	**<** **0.001**
Discharge echocardiography^d^*, *mean ± SD					
– Aortic valve area (cm^2^), median (IQR)	1.80 (1.50–2.20)	1.60 (1.40–2.07)	1.80 (1.50–2.20)	2.00 (1.60–2.30)	**<** **0.001**
– Mean aortic gradient (mm Hg), median (IQR)	9.0 (7.0–13.0)	9.0 (7.0–12.0)	9.0 (7.0–13.0)	10.0 (7.0–14.0)	0.20
– AR ≥ moderate, *n* (%)	127 (11)	41 (11)	46 (10)	40 (12)	0.73
– MR ≥ moderate, *n* (%)	211 (19)	53 (16)	95 (21)	63 (19)	0.21

*AV* atrioventricular, *IQR* interquartile range, *AR* aortic regurgitation, *MR* mitral regurgitation

^a^No percentage since cohort 3 did not complete the full 1‑year follow-up

^b^No percentage since cohorts 2 and 3 did not complete the full 3‑year follow-up

^c^Percentage is based on number of patients with new pacemaker divided by number of patients without a pacemaker at baseline

^d^Data not available in approximately one-third of the total cohort

While age and gender did not change from T1 to T3 (80, 80 and 79 years and 53%, 55% and 52% male gender, respectively), the patient’s baseline risk (i.e. Society of Thoracic Surgeons (STS) risk score) dropped from 4.5% to 2.7% (*p* < 0.001) due to a progressive decline in the prevalence of antecedent cardiovascular disease (i.e. myocardial infarction, coronary bypass surgery and peripheral artery disease) (Electronic Supplementary Material, Fig. S1). In addition, the aortic valve area at discharge increased significantly over time (1.60–2.00 cm^2^, *p* < 0.001). There was a slight, but significant, increase in the prevalence of atrial fibrillation (31%–35%). The prevalence of hypertension and renal failure fluctuated.

**Fig. 1:**
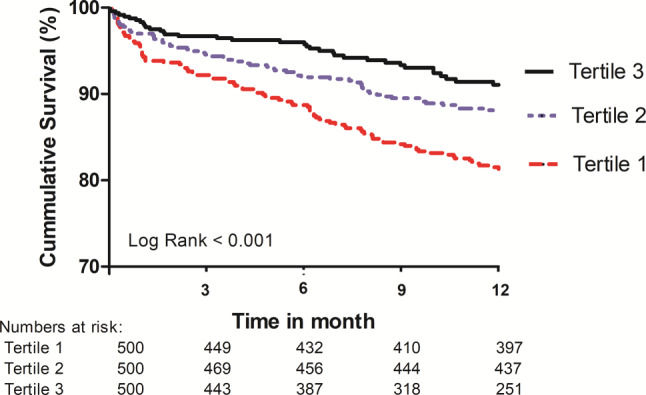
Overall 1‑year survival

With respect to the TAVI procedure, general anaesthesia was virtually eliminated (100%, 24% and 1% from T1 to T3). Transfemoral TAVI remained the default approach (87%, 94% and 92%) with subclavian being the most common alternative (1%, 0% and 7%) (Electronic Supplementary Material, Fig. S2). Also, there was a decrease in the use of balloon pre-dilatation (from 84% to 25%, *p* < 0.001), albeit with a higher overall need for balloon post-dilatation (19%–30%, *p* < 0.001). Initially, the self-expanding prosthesis CoreValve (Medtronic, Minneapolis, MN, USA) was the only valve used (later replaced by the Evolut R and Evolut PRO). In 2007, the balloon-expandable Edwards Sapien3 valve (Edwards Lifesciences, Irvine, CA, USA) was introduced into our programme, followed by the mechanically expanded LOTUS valve (Boston Scientific, Marlborough, MA, USA) in 2013. Vascular closure was predominantly performed surgically in T1 (78%), while a fully percutaneous approach was used in T3 (97%, *p* < 0.001). Following its introduction in 2013, the use of filter-based cerebral embolic protection (CEP) increased from 26% to 43% (*p* = 0.001). Overall, the median procedure time and contrast volume reduced significantly (186, 114 and 56 min and 120, 100 and 80 ml, respectively). With respect to outcomes, the frequency of 30-day all-cause mortality (7%, 4% and 2%), all stroke (7%, 3% and 3%), new pacemaker implantation (19%, 22% and 8%) and delirium (17%, 12% and 8%) improved significantly. The frequency of major bleeding and vascular complications did not change over time (both approximately 9%, 6% and 6% over the entire study period). One-year survival increased significantly from T1 to T3 (Fig. 1).

## Discussion

Over our 15 years’ experience, we found that the age of patients who received TAVI in our institution (approximately 80 years) did not change, while operative risks dropped from prohibitive/high to intermediate, mainly because of fewer antecedent cardiovascular diseases. In conjunction, the in-hospital complication rates and 1‑year mortality improved significantly. Another finding is the exponential growth in TAVI that preceded the publication of the two RCTs assessing the role of TAVI in intermediate-risk patients (2016) [14, 15]. This increase likely reflects the embracement of TAVI by the patients and their relatives, as well as by the medical professionals and institutions, based upon the minimally invasive nature of the procedure (including local anaesthesia) and its sound technical/physiological concept, namely the effective reduction of increased afterload of the left ventricle by the replacement of a stenotic valve similar to surgical replacement.

The reason for improved outcomes is obviously multifactorial and the question as to which factor played a (more) prominent role cannot be answered given the observational nature and time bias of this study. Patient-specific factors but also growing operator experience, refinements in catheter and device technology and procedure simplification will have played a role in reducing risks in favour of better outcomes. These advancements likely played a role in achieving a greater aortic valve area and overall valve performance over time, which is known to favourably influence outcomes. Simplification measures (i.e. abandoning the use of general anaesthesia, use of a femoral approach in almost 100% of cases) likely resulted in significant reductions of delirium and mortality [12]. It has to be acknowledged that the present series stems from an institution that was an early adopter of TAVI at a time when TAVI was still in an experimental phase with limited experience worldwide, during which general anaesthesia, surgical cut-down and circulatory support were the standard, in addition to the fact that only patients at extreme risk were considered as candidates [16]. Also, the institution played a role in the elimination of circulatory support and the introduction of echo-guided access with a percutaneous closure technique that paved the way for a true percutaneous approach in TAVI [17].

It may be assumed that the combination of the factors described above plus the implementation of TAVI in the true low-risk or so-called surgical candidate will further reduce complication rates and improve outcomes. The incessant improvement of outcomes over time is illustrated by the RCTs that initially recruited extreme-risk (2010) [2, 3] and subsequently intermediate-risk (2017) [14, 15] and most recently (2019) [9, 18] low-risk patients. It is noteworthy that data from the PARTNER III and NOTION trials have contributed to expanding indications for TAVI in the updated European Society of Cardiology guidelines for valvular heart disease, supporting the selection of lower-risk patients based on multidisciplinary heart team consensus [8, 9, 19].

At the commencement of the TAVI programme at our centre, the function and position of the heart team was elementary and foundational in nature. The team initially comprised only a cardiologist and a cardiothoracic surgeon, whereas since 2010 discussions on structural heart disease cases are held on a regulated, weekly basis, using a formalised, multidisciplinary approach, involving cardiologists, surgeons and geriatricians. With the inclusion of geriatricians in the heart team, more specific attention is paid to patient perspectives and preferences, coupled with life expectancy and the influence of the frailty status (i.e. utility vs futility) [10, 20–22].

As we learned by doing and as it became clear that ‘fixing the heart’ is just one part of the management of the—in general elderly—patient with cardiac disease, the role of the geriatrician became pivotal and led to the creation of the TAVI Care & Cure programme and patient pathway [11], a clinical and scientific programme that started in October 2013 and is approved by the medical ethics committee of the Erasmus MC. All patients referred for TAVI are seen at the outpatient clinic of the Departments of Cardiology and Geriatrics, where pre-defined clinical variables are collected that are entered into the electronic medical record and anonymously into a dedicated research database. The main clinical objective was to further improve patient selection by a more refined benefit/risk prediction via a comprehensive patient evaluation followed by the above-mentioned multidisciplinary discussion [11].

During the same time, a rapid transition of TAVI using general anaesthesia to TAVI under local anaesthesia was instituted (first local anaesthesia September 2012) [23, 24]. Other changes in the execution of TAVI were, in chronological order, the use of filter-based CEP beginning in January 2013 and plug-based arterial closure techniques (first application 23 July 2015) as well as the sutured-based techniques, with the easier-to-use Proglide replacing the more complex Prostar closure system (both Abbott Vascular, Santa Clara, CA, USA) [25–28]. These changes over time—in addition to improved treatment planning via heart team discussions, experience, and refinement in technology—may have contributed to better outcomes. However, such changes may actually confound outcome in the opposite direction, since their introduction implies a learning curve and, thus, risk upon initiation. Furthermore, notwithstanding the fact that CEP effectively captures debris travelling to the brain during TAVI, only indirect evidence suggests a reduction in stroke incidence [29, 30]. Currently, the Achilles’ heel of TAVI remains new conduction abnormalities and the eventual need for a new permanent pacemaker (PPM). We observed a significant reduction in PPM implantation from 22% to 8% from T2 to T3. The question is whether this is a chance finding or a real improvement due to a combination of more appropriate valve selection (e.g. avoidance of mechanically expanded valves in patients at risk of new conduction abnormalities), improved implantation technique (i.e. operator experience) or post-operative management, e.g. by prolonged monitoring instead of quick discharge and low PPM threshold [31–33]. Although the frequency of pre-existing bundle branch block has remained stable over time, it is conceivable that the lower-risk profile of patients in T3 as compared to T2 (STS score 4.3 vs 2.7, *p* < 0.001) played a key role in the lower frequency of PPM in T3. This is in line with the findings of the PARTNER cohort B trial, in which pacemaker rates were equal irrespective of wether TAVI was performed or not [2]. Also, mechanically expanded prostheses were used less often in T3 as compared to T2, which may in part explain the lower need for pacemakers in T3. In the present series we found no decrease in moderate to severe aortic regurgitation post-TAVR. As experience has invariably increased over time, the absence of a reduction in aortic regurgitation post-TAVR may be related to specific patient-related factors, such as valve anatomy or reduced use of mechanically expanded prostheses—known to be associated with the least amount of residual aortic regurgitation—in T3 [34].

What we have learned from looking back at our data spanning a period of 15 years of experience with TAVI is the pivotal role of innovation. Yet, this comes with a responsibility, since one should not offer treatment because it is available but because the patient is expected to benefit in terms of survival and/or quality of life. This delicate balance (survival or quality of life) depends on a set of variables that go beyond the standard and easy-to-collect cardiovascular variables and must include a more comprehensive or holistic approach, for which medical specialists such as geriatricians are essential, as they have an understanding of the patient and his/her disease and, hence, the outcome of treatment from a much broader perspective. All too often we have learned that, despite successful treatment, life expectancy or independence of daily living or quality of life were lower than expected. Since 2013, the geriatricians have been playing an active and primordial role in treatment decision making during the heart team discussions, to avoid futility and promote utility.

### Limitations

The objective was to report changes in baseline characteristics of patients treated with TAVI since the initiation of our programme in 2005. In addition, we have reported changes in the procedure and also reported on in-hospital outcome and 1‑year mortality. With respect to the primary objective, observational bias may have played a role, in particular in the period before the start of the TAVI Care & Cure project. The latter was characterised by the collection of pre-defined variables related to patient characteristics and outcomes via a study protocol approved by the medical ethics committee. Observation bias may also have played a role in the in-hospital outcomes but not in the assessment of mortality, including during follow-up, for which the Dutch Civil Registry was used. The main limitation may be the tertiary referral nature of our institution (generalisability) and the fact that we cannot directly relate outcomes to changes in patient-related, procedure-related or operator-related factors (time bias). With the simplification of the TAVI pathway and a reduction in complication risks, total hospital admission times likely declined over time, although the data confirming this hypothesis were incomplete in this study.

## Conclusion

We found that since the initiation of TAVI at our institution in 2005, the age of patients treated with TAVI did not change. Yet, their baseline risk profile improved mainly because of fewer antecedent cardiovascular diseases. Therefore, the patients treated with TAVI remain primarily octogenarians but at intermediate surgical risk. TAVI was associated with significant improvement of outcomes and 1‑year survival. Bleeding and vascular complications as well as the need for a pacemaker remain the Achilles’ heel of TAVI.

## Supplementary Information

**Electronic Supplementary Material, Fig. S1** Median (interquartile range) STS score and mean (standard deviation) age stratified per tertile

**Electronic Supplementary Material, Fig. S2** Assessment of access routes for TAVI in the Erasmus MC
