# Supplementary figures and images for: Changes in demographics, treatment and outcomes in a consecutive cohort who underwent transcatheter aortic valve implantation between 2005 and 2020
Source: Neth Heart J. 2022 Feb 25;30(9):411–22. doi: 10.1007/s12471-022-01662-2 (PMC9402869; doi:10.1007/s12471-022-01662-2)

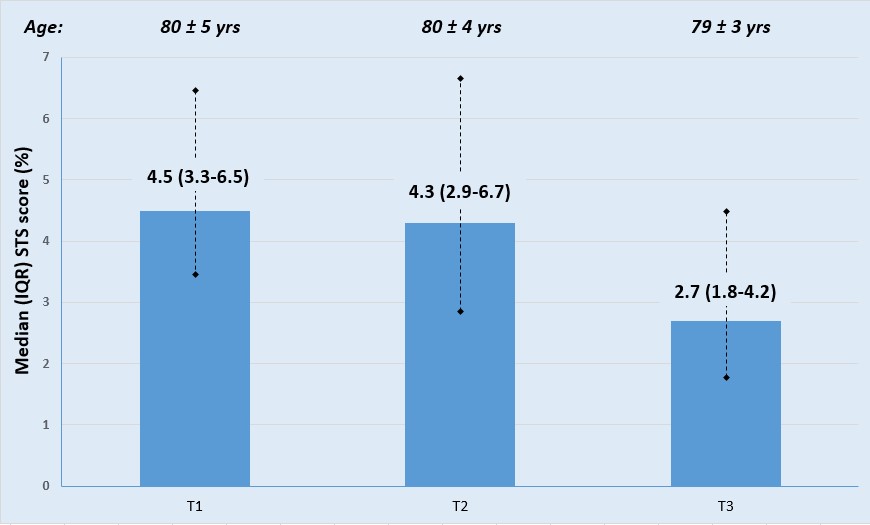

Supplement: Supplementary file 1 — Electronic Supplementary Material, Fig. S1 Median (interquartile range) STS score and mean (standard deviation) age stratified per tertile [file 12471_2022_1662_MOESM1_ESM.jpg]

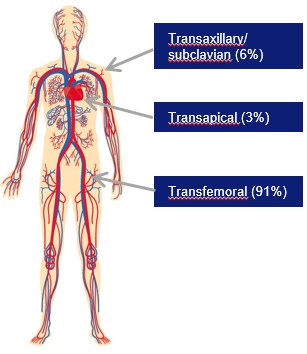

Supplement: Supplementary file 2 — Electronic Supplementary Material, Fig. S2 Assessment of access routes for TAVI in the Erasmus MC [file 12471_2022_1662_MOESM2_ESM.jpg]
